# A Pre-Column Derivatization Method for the HPLC-FLD Determination of Dimethyl and Diethyl Amine in Pharmaceuticals

**DOI:** 10.3390/molecules29235535

**Published:** 2024-11-23

**Authors:** Georgios Kamaris, Maria Tsami, Georgiana-Roxana Lotca, Sofia Almpani, Catherine K. Markopoulou

**Affiliations:** Laboratory of Pharmaceutical Analysis, Department of Pharmacy, Aristotle University of Thessaloniki, 54124 Thessaloniki, Greece; kamarisg@pharm.auth.gr (G.K.); tsamimaria@gmail.com (M.T.); grlotca@gmail.com (G.-R.L.); salmpan@pharm.auth.gr (S.A.)

**Keywords:** HPLC-FLD, diethylamine and dimethylamine, NBD-Cl, pre-column derivatization, SPE, pharmaceuticals

## Abstract

In recent years, the detection of nitrosamine precursors has become an important issue for regulatory authorities such as the European Medicines Agency (EMA) and the Food and Drug Administration (FDA). The present study provides a pre-column derivatization method for the analysis of dimethylamine (DMA) and diethylamine (DEA) in pharmaceutical products using HPLC and a fluorescence detector. Appropriate chromatographic parameters, including mobile phase composition (organic solvent, buffer, pH), elution type, flow rate, temperature, and λexcitation/emission, were investigated. Analysis was performed at λ_excitation_ = 450 nm and λ_emission_ = 540 nm on a C_18_ column (at 40 °C) using gradient elution as a mobile phase with Eluent A: Phosphoric Acid Buffer (20 mM, pH = 2.8) and Eluent B: methanol, with a flow of 0.8 mL/min. The method was validated according to ICH specifications in terms of linearity (0.5–10 ng/mL for DMA and 5–100 ng/mL for DEA), specificity, and robustness, as well as repeatability, intermediate precision (%RSD < 2.9), and accuracy (% recovery 98.2–102.0%). The derivatization process was optimized using the “Crossed D-Optimal” experimental design procedure, where one mixture component was cross-correlated with two factors. The stability of the samples was studied over a period of one month. To process the samples (pharmaceuticals), various purification techniques were tried using solid/liquid or liquid/liquid extraction with dichloromethane. Finally, a straightforward solid-phase extraction (SPE, C_18_) method was chosen prior to derivatization. The method was successfully applied, since the extraction recoveries were >81.6% for DMA (0.5 ppm) and >81.1% for DEA (5 ppm). Based on the results obtained and the available literature, the scientific community seeks, by proposing flexible analytical methods, to delimit the problem of nitrosamines.

## 1. Introduction

Diethylamine (DEA) and dimethylamine (DMA) are secondary aliphatic amines featuring two methyl or ethyl groups as N-substituents, respectively. Both can be present in pharmaceutical formulations or Active Pharmaceutical Ingredients (APIs), more often as degradation products originating from the drug substance itself rather than as impurities from its synthesis process [1].

In pharmaceuticals, DMA plays an important role in the production of drugs such as metformin (precursors: 2-cyanoguanidine and dimethylamine hydrochloride) and acetyl-dimethylamine (a combination of dimethylamine and acetic acid). In addition, it is a fundamental ingredient in the preparation of myristyl alcohol, a synthetic compound derived from acetaldehyde and dimethylamine. Last but not least, and outside of the pharmaceutical realm, dimethylamine serves as a solvent to create the elastic polymer found in fabric [1]. Diethylamine serves various purposes in the chemical industry, including its role as an intermediate for producing the corrosion inhibitor N,N-diethylethanolamine, contributing to pesticide and insect repellent manufacturing, as well as participating in the processing of rubber polymers. Regarding pharmaceutical products, it has been used as an excipient in topical pharmaceutical preparations with anti-inflammatory action (such as acetylsalicylic acid or diclofenac) because it enhances transdermal absorption [2,3] A recent research study showcased diethylamine as an active pharmaceutical ingredient (API) with analgesic properties that is utilized in pharmaceutical formulations. Lastly, DEA also serves as a precursor in the synthesis process of some drugs, such as lidocaine [2].

However, despite the wide range of applications, their use should not be thoughtless due to their toxicity. An overview of 12 toxicity properties related to DMA and DEA, calculated with PreADMET software (https://preadmet.webservice.bmdrc.org/, accessed on 25 October 2024) [4] (Table S1), showed that both substances have carcinogenic properties, with DEA presenting additional mutagenicity.

The toxicity of the two substances is also indirect, since they contribute to the synthesis of nitrosamines through various mechanisms. Dimethyl and diethylamide are sensitive to oxidation, resulting in the formation of asymmetric dimethyl/ethyl hydrazine and, subsequently, N-nitrosodimethylamine (NDMA) and N-nitrosodiethylamine (NDEA), which have been classified by the US Environmental Protection Agency as “potentially carcinogenic to humans”. Finally, and according to the World Health Organization’s International Agency for Research on Cancer, cytochrome P450 activates DMA and DEA, resulting in the creation of NDMA and NDEA. The nitrosamines then decompose, creating diazonium and carbene ions, which act as active alkylating agents capable of modifying DNA [4].

Taking into account the side effects of both substances, the importance of their detection and quantification in pharmaceutical preparations becomes evident. However, HPLC-UV determination methods of DMA and DEA present difficulties due to their low absorption in UV radiation [5]. Moreover, considering their small size and high polarity, they exhibit poor retention in reversed-phase columns. Therefore, an efficient HPLC analysis approach involves pre-column derivatization, which leads to the generation of more hydrophobic and strongly absorbing UV/fluorescence derivatives. The official pharmacopeial technique for the ideal determination of DMA and DEA requires an extended derivatization procedure to attain favorable chromatographic characteristics [6]. Alternative methods include gas chromatography (GC), which, however, also presents concerns, like prolonged analysis times and relatively high limits of quantification [7]. These analytical challenges identify the necessity of the development of more robust and sensitive analytical methods, such as those using fluorometric detectors [5].

Currently, there are an increasing number of publications reporting the analytical methodologies being developed on the basis of derivatization in the field of chemical analysis [8]. In general, it is imperative to ensure the reproducible or quantitative nature of the derivatization reaction in both standards and analysis assays. The decision between pre- and post-derivation depends on factors such as the chemical properties of the derivatizing reagent relative to the target analyte [9]. Usually, off-line pre-column derivatizations offer advantages, including no extra-column loss of flexibility or efficiency in reaction conditions, as well as optimization for high reaction yields. However, attention and manipulation are needed [10].

Numerous derivatization methods have been specifically developed for amine groups in substances, employing reagents like o-phthalaldehyde (OPA), dansyl-Cl, Fmoc-Cl, and Marfey’s reagent [11].

In 1968, Ghosh and Whitehouse introduced 7-Chloro-4-nitrobenzo-2-oxa-1,3-diazole (NBD-Cl), a reagent which can interact with several chemical groups (4–5). NBD-Cl is produced through the nitration of 4-chlorobenzofurazan, derived from 2,6-dichloroaniline using dichloro-nitrosobenzene [12,13]. The reagent is widely employed as a fluorogenic mostly in the examination of amino acids, primary or secondary amines, thiols, and lipids. Its chemical structure makes it applicable in various analytical chemistry contexts [12,13]. Researchers have explored various modifications and substitutes for NBD-Cl, with the goal of reducing reaction times and increasing derivatization yields. Despite continuous developments, NBD-Cl remains a versatile and widely used fluorogenic reagent, prompting scientists to explore its variations to enhance its effectiveness in numerous applications [10].

Several studies have also focused on the detection of dimethylamine (DMA) and diethylamine (DEA) after a derivatization process on different substrates, using HPLC and a fluorescence detector (FLD). Noteworthy is the case of their analysis in water samples using OPA and NBD-Cl as derivatization reagents. OPA was used to remove interferences from primary aliphatic amines, while NBD-Cl was used to selectively react with secondary amines [14]. Another study delves into the examination of primary and secondary amines (including DMA) in biological fluids using HPLC with an FLD detector. For the derivatization, the reagent 1,3,5,7-tetramethyl-8-(N-hydroxysuccinimidylbutyrate)-difluoroboradiase-s (TMBB-Su) was used [15]. Similarly, the detection of dimethylamine in pharmaceutical formulations has also been investigated using ion exchange chromatography (cation exchange for DMA and anion exchange for nitroso) with UV detection [16]. An alternative method for the determination of the two substances without derivatization would be the MS/MS detector. However, the instrumentation is expensive, and difficulties are generally encountered in the detection of low-molecular-weight substances.

The present study aims to establish a reliable, sensitive, and flexible derivatization technique for the analysis of both DMA and DEA in pharmaceutical products using HPLC-FLD. The developed method involves three key stages: (a) pre-column derivatization using the 4-chloro-7-nitrobenzofurazane (NBD-Cl) reagent in the optimized conditions; (b) establishing a final separation and quantitative determination method for the two amines using HPLC-FLD; and, last but not least important, (c) the application of a new, reliable, and easy-to-use SPE technique for sample purification that can be used on complex pharmaceutical substrates.

## 2. Results and Discussion

### 2.1. Derivatization Procedure

Monitoring the reproducibility of a derivatization reaction is essential, ensuring consistent results at the end of an assay [17] Pre-column derivatization provides the advantages of using simple equipment (for large-scale reactions) and generating chromatograms that do not require further modifications.

However, a central aspect of this research was the optimization of the reaction steps to make the process simple and efficient on any substrate. The reaction conditions, such as its stoichiometry and the chemical structure of the product, initially played the main role. Subsequently, critical parameters such as product stability, solvents used, temperature, pH, and reaction time were carefully selected [11,18] after investigation.

#### 2.1.1. Chemical Reaction

In a preliminary study, two derivatization reagents, Dansyl-Cl and NBD-Cl, were evaluated, with the latter showing higher chromatographic signals for both DMA and DEA and thus being selected for the reaction. NBD-Cl is categorized within the substituted benzooxadiazoles group. Due to its properties, it engages with aliphatic proteins, peptides, amino acids, and amines. On the other hand, its reactivity with thiols or phenols under alkaline conditions is diminished [19].

**Figure 1:**
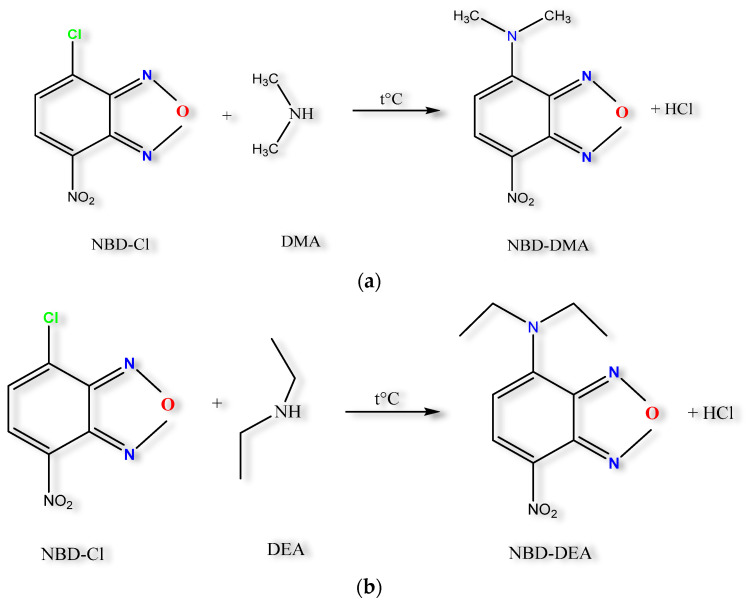
Reaction between NBD-Cl and (**a**) DMA, (**b**) DEA.

Particularly for secondary amines, the reaction with NBD-Cl can be carried out in an aqueous or organic solvent. When NBD-Cl is dissolved in methanol, it undergoes partial solvolysis, resulting in the formation of NBD-OCH3, which facilitates [20] the process, since the highly reactive chlorine atom (from the structure of NBD-Cl) is replaced faster with the DMA and DEA moieties (Figure 1a,b) [21]. According to the literature, the ideal pH at which the reaction should take place is between 8–11 [9,10] Boric acid can be used as a buffer solution to adjust the pH range from 7.8 to 10.6. In a basic environment, at an appropriate temperature, and over the necessary time, the reaction is completed when the compound changes from colorless to yellow-orange. The derivatized products display vibrant fluorescence characteristics in contrast to NBD-Cl, which lacks fluorescence ability [10].

#### 2.1.2. Temperature

Increasing the temperature often enhances the yield of the derivatization and shortens the reaction time, while the combination of stirring and heating can further accelerate the overall reaction time and maintain reproducibility.

In the present study, different temperatures (40 °C, 70 °C, 80 °C, and 100 °C) were investigated, aiming to determine the optimal one. In all cases, after the completion of the reaction, the sample was placed in the freezer for 1 min to terminate the process. At 100 °C, part of the sample evaporated (even though the container was hermetically sealed), and therefore, this temperature was discarded. The results showed that optimal performances were achieved at 70 °C and 80 °C, and the temperature of 70 °C was chosen to reduce the chances of sample evaporation or damage (Table S2). Consequently, the derivatization mixture was placed on a hot plate with magnetic stirring, reaching a temperature of 70 °C for 30 min (optimal reaction time).

#### 2.1.3. Diluents

Methanol is the suitable solvent for NBD-Cl, while water is recommended for the borate buffer solution. Regarding the solubility of the two amines, both solvents were sufficient, with water being superior (DMA: 136 g/100 mL H_2_O, DEA: 104 g/mL H_2_O) over methanol. Therefore, in order to achieve the optimal result, a further study was carried out using various solvent mixtures, such as H_2_O/MeOH or NaHCO_3_ (0.5 M, pH = 7)/MeOH, in various proportions, while the rest of the experimental conditions were kept constant. The presence of NaHCO_3_ was intended to deprotonate the two amines in a preliminary stage in order to facilitate the alkylation that followed [22]. Based on the results, it was found that the ratio of 50:50 *v*/*v* methanol–water (with or without NaHCO_3_) gave the largest relative signal, but since the differences were not significant, to simplify the method, it was decided to use only water.

#### 2.1.4. Borate Buffer Incorporation

With the primary objective of improving the efficiency of the reaction but also facilitating the process, research was conducted on how to incorporate the borate buffer solution. Therefore, two distinct procedures were performed: In the first, buffer was added into the stock standard solution of DMA and DEA. In contrast, in the second approach, borate was prepared separately and added to the derivatization reaction along with the other two reagents (NBD-Cl and DMA/DEA). Following the measurement of the two samples and subsequent analysis of the results, it was observed that the second approach produced superior outcomes (AUC_DMA(1st)_ = 395,419 and AUC_DMA(2nd)_ = 518,209; and DEA, AUC_DEA(1st)_ = 221,379 and AUC_DEA(2nd)_ = 321,945).

#### 2.1.5. pH of Borate Buffer Solution

pH plays a critical role in the derivatization reaction, affecting the balance between neutral and protonated amines in a solution. A higher concentration of neutral amines enhances the availability of substances for reaction with the derivatization reagent and subsequent derivatization. Considering the pKa values for both DMA and DEA (10.7 and 11.1 respectively), pH values of 9.0, 10.0, and 11.0 were examined. Based on the results (Figure S1), pH = 11.0 yielded higher AUC values, especially for the DEA peak, and thus was adopted for the experimental procedure.

#### 2.1.6. Concentration of Borate Buffer Solution

The concentration of the boric acid in the buffer solution significantly affects the outcome of the derivatization process. Acting as a weak acid, boric acid creates an equilibrium with its conjugate base (borate ion), forming a buffer solution. The buffering capacity, indicative of the solution’s ability to resist changes in pH, depends on the ratio of the weak acid to its conjugate base. Increasing the concentration of boric acid predicts a shift of the equilibrium towards an increased concentration of the boric ion, increasing the buffering capacity. For amines, maintaining a constant pH is paramount to the success of the reaction.

To explore the effects of different concentrations, three levels of borate buffer (5 mM, 10 mM, and 20 mM) were tested. Higher concentrations were avoided so as not to overload the column. The results (Figure S2) revealed that variations in boric acid concentration significantly affected the area of peak (AUC) of the analytes, especially for DEA. Therefore, the highest concentration (20 mM) was chosen as the optimal one for the experimental procedures.

#### 2.1.7. Concentration of NBD-Cl

To ensure the sufficiency of NBD-Cl in the reaction, 3 concentration levels of 1.0 mg/mL, 0.33 mg/mL and 0.15 mg/mL were tested, respectively. For the test, 150 µL of these solutions were transferred separately to a total sample volume of 400 µL to perform the derivatizations. Based on the results, it was found that high concentrations of NBD-Cl yielded the largest peak areas (1.9 times for DMA and 3.1 for DEA) for both derivatives. However, although the increase in signal was significant, additional peaks were observed in the blank, co-eluting with DMA. Furthermore, when a large amount of derivatization reagent was used, a double peak of DEA appeared. For these reasons, 0.33 mg/mL was chosen as the optimum concentration of NBD-Cl.

#### 2.1.8. Crossed D-Optimal Experimental Design Methodology

Experimental design methodology (Design Expert 11, software) was applied to determine the remaining factors that could affect the reaction efficiency [23]. Therefore, based on the Crossed D-Optimal Experimental Design (CDO) technique, the volumes of the reagent solutions (VmL NBD-Cl and the aqueous solution of DMA, DEA) were combined with two factors (VmL borate buffer and the reaction time) and were determined (Table 1). A total of 18 experiments were required to evaluate these parameters.

Given the results of the analysis of variance (Table 2a,b), changes in the component content (A: Volume of NBD-CL, B: Volume of DMA/DEA aqueous solution) as well as in factor values (C: Volume of borate buffer, D: reaction time) were considered significant (*p*-value < 0.05) and affected the two responses (AUC of DEA and DMA). Thus, based on the developed mathematical models, the equations for calculating responses for both DEA and DMA were formulated as a function of all of these factors (Table S3). Moreover, the R-squared values were found to be >0.9729, confirming the reliability of the model, while the values of the predicted R-squared agreed with those of the adjusted R-squared. Finally, the value of the ratios indicated sufficient signal-model discrimination (Adequate Precision > 4).

In the plots of the residuals against the predicted values of DMA and DEA (Figure S3A), their random dispersion in a constant range on either side and along the predictions on the X-axis becomes evident. Accordingly, in the graph of the actual response values against the predicted (Figure S3B), correlations are represented by a straight line.

The optimal values for conducting the experiment were calculated from Derringer’s desirability function (value 0.917). According to the equation, the reaction is completed in 29 min, while the volumes of the reagent solutions are 50 µL buffer H_3_BO_3_ (20 mM), 150 µL NBD-Cl (0.33 mg/mL), and 200 µL DMA + DEA in H_2_O. Thus, the derivatization reaction, based on the results of the experimental design and preliminary experiments, is formulated as follows:

Into a small glass vial (with a flat base surface), 200 μL of DMA + DEA aqueous solution, 50 μL of borate buffer (20 mM), and 150 μL of NBD-CL (0.33 mg/mL) were quantitatively transferred and mixed, by vortex, for 1 min. The sample was then placed in a heating block at 70 °C for 30 min and in the freezer for 1 min to stop the reaction (Figure S4). After the completion of the reaction, the sample was analyzed by HPLC.

#### 2.1.9. Stability Control of the Derivatized Product

**Figure 2:**
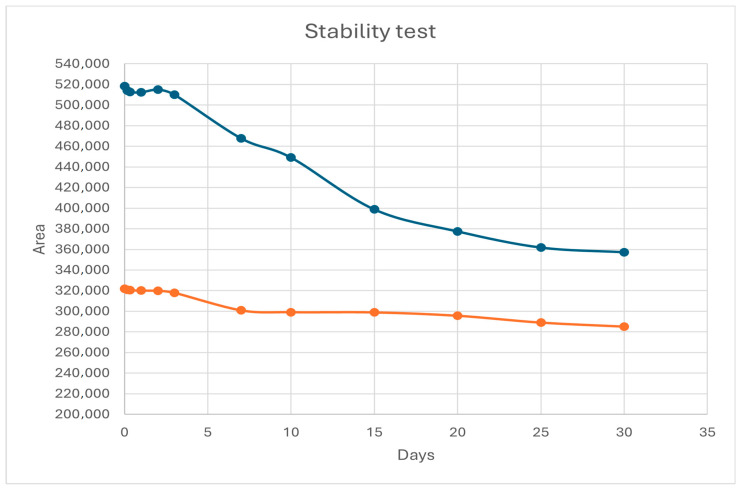
Stability test for NBD-DMA (blue) and NBD-DEA (orange) derivatives.

In order to check the stability of the NBD-DMA and NBD-DEA complexes, their recoveries were calculated at regular time intervals for one month. In the interim, the samples were stored at 2–8 °C. According to the results diagram (Figure 2), both complexes remained stable for at least the first two days, while after one month, they showed recovery rates of 69.5% for DMA and 88.8% for DEA.

### 2.2. Mobile Phase Composition

By investigating the HPLC analytical method, various chromatographic parameters were studied. Regarding the mobile phase, different methanol/water mixtures of 20–60% *v*/*v* were tested, and 50% *v*/*v* (at isocratic conditions) was chosen as the optimal ratio. In addition to methanol, acetonitrile also gave similar chromatograms (methanol: Tf_DMA_ = 1.05 and Tf_DEA_ = 1.08 and resolution > 2.13, acetonitrile: Tf_DMA_ = 1.33 and Tf_DEA_ = 1.14 and resolution > 3.21), but was rejected due to its relatively high cost.

For the aqueous solvent of the mobile phase, it was observed that replacing water with a buffer solution improved the shape of the peaks. Phosphate [24], ammonium acetate [25], and sodium acetate [24] buffers have been recommended in the literature for amino acid analysis. Phosphate buffer has significant buffering capacity and provides a low background signal with reproducibility. Aqueous solutions of formic acid also exhibit similar properties [24]. Therefore, experiments were conducted to evaluate the feasibility of using formic acid or phosphate buffer in the mobile phase. After comparing the chromatograms (Figure S5), it was evident that neither case showed a significant signal difference (2.0% peak area difference for DMA and 0.5% for DEA), nor was there a difference in the retention times of the two substances. A significant difference was observed only in an interfering peak eluting before DMA. In the case of formic acid, this peak eluted much closer to DMA, reducing the separation capability of the system. Therefore, phosphate was chosen as the most suitable buffer in the mobile phase.

Regarding the pH determination, three different values lower than the pKa of the amines (DMA = 10.7 and DEA = 11.1) were considered: 2.8, 3.5, and 5.5. In all situations, the system exhibited the same separation capacity and similar chromatographic peak behaviors. The pH value of 2.8 was chosen as optimal, as it ensured the stability of the derivatives for both substances. The next step involved investigating the optimal concentration of NaH_2_PO_4_ buffer from 20 to 50 mM (pH 2.8). Based on the results, no significant differences were observed, and therefore, the lowest concentration (20 mM) was chosen to prevent column overload. An additional problem that had to be addressed was cleaning the column at the end of each injection. Therefore, a gradient elution system was applied where the content of the organic solvent gradually increased (Eluent A: NaH_2_PO_4_, 20 mM and Eluent B: MeOH). Finally, the study of the appropriate injection volume was carried out in the range of 10–80 µL at two concentration levels of the amines (low: 0.5 ng/mL DMA and 5 ng/mL DEA; high: 10 ng/mL DMA and 100 ng/mL DEA). For signal amplification, 80 µL was chosen as the optimal injection volume. To select the appropriate emission and excitation wavelengths (λ) of DMA and DEA, the FLD detector scanned the sample at zero flow rate of the mobile phase (Figure S6) [10,12,18]. The results obtained showed that wavelengths of 470 nm (excitation) and 540 nm (emission) produced the strongest signals.

### 2.3. Method Validation

The validation of an analytical method is a pivotal prerequisite for its application. Key parameters, according to the guidelines by the International Conference on Harmonization (ICH, Q_2, R1, Validation of Analytical Procedures) [26], include specificity, linearity, repeatability, intermediate precision, accuracy, and robustness.

#### 2.3.1. Specificity

**Figure 3:**
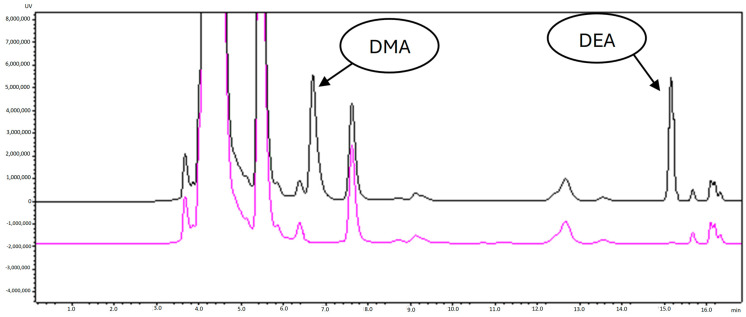
Chromatograms of blank (pink) and diluted standard mix (black) of DΜA and DΕA (C_DMA_ = 10 ng/mL and C_DMA_ = 100 ng/mL).

In theory, specificity implies the ability of a method to identify the analytes of interest even when other components are present in the sample, such as impurities, degradation products, or related substances. Therefore, in the present conditions, two samples were prepared and analyzed: a blank (150 µL NBD-CL, 50 µL borate buffer and 200 µL H_2_O) and a standard containing 150 µL NBD-CL, 50 µL borate buffer, and 200 µL of standard mixture of DMA (0.5 ng/mL) and DEA (5 ng/mL). When comparing the two chromatograms (Figure 3), no additional peaks were observed in the elution times of the two complexes, NBD-DMA (~7.5 min) and NBD-DEA (~15 min).

#### 2.3.2. Linearity, Limit of Detection (LOD), and Limit of Quantitation (LOQ)

Linearity should be assessed by visual inspection of a plot of signals as a function of analyte concentration or content. Therefore, six standard solutions of different concentrations for each amine (diethylamine and dimethylamine) were prepared to plot the calibration curve, and then analyzed in duplicate. From the obtained data, standard calibration curves were calculated by the least-squares method (Table 3).

#### 2.3.3. Accuracy

The precision of an analytical method is expressed as % recovery of the analyte in the sample. In the present study, five mixed samples (spiked) with known concentrations of the two amines were produced and re-determined. Table 4 shows the % recoveries of dimethylamine and diethylamine.

According to the results, the recoveries of dimethylamine and diethylamine were satisfactory, proving that the method is reliable.

#### 2.3.4. Intra- and Inter-Day Precision

The intra-day repeatability of DMA and DEA was calculated by applying three replicates at three levels of their concentrations based on the relative standard deviation % RSD. Likewise, for inter-day repeatability (three replicates), samples were studied at the same concentration levels on three consecutive days (inter-day) (Table 5).

#### 2.3.5. Robustness

To assess the robustness of the method, small modifications (±1) were made to the column temperature, the flow rate, λ em/ex, and the mobile phase composition (Table S4). Examining the % RSD values of the peak areas and tailing factors for both substances, as well as the resolution factor with the nearest interfering peak, it was observed that changes in the parameters had minimal effects on the results. Therefore, the method is considered robust.

### 2.4. Sample Extraction Procedure

Since the proposed method was validated, the quantification of DMA and DEA followed in two pharmaceutical compositions (Uniphyllin^®^ (Uni-pharma, Athens, Greece), Xylozan^®^ (Demo, Athens, Grecce)) containing theophylline and lidocaine as APIs, respectively. Due to the complexity of the substrate, a stage of pretreatment and purification of the samples preceded the test. Therefore, two extraction procedures were studied.

#### 2.4.1. Liquid Extraction (LE)

In the liquid extraction process, it was crucial to use a solvent that selectively dissolved the amines, separating them from the matrix without affecting their stability. For this reason, dichloromethane was used as the extraction eluent for all samples. In the solid formulation (Uniphyllin^®^), pulverization was performed to reduce the particle size, increasing the solid–liquid interface. After grinding, approximately 25.0 mg of solid or liquid formulation (Xylozan^®^) was accurately weighed (in duplicate) into two 15 mL Eppendorf tubes. One of the two samples was spiked with 50 μL of dichloromethane solution containing mixed STD1 of DMA (60 ng/mL) and DEA (600 ng/mL), while the other contained 100 μL of dichloromethane (blank). At the same time, a third sample that did not contain the substrate was spiked with 100 μL of mixed STD1 and subjected to the same treatment as the rest of the samples. All samples were subjected to purification using the most efficient method, which emerged after much research (Figure S7) and included the following steps: 10 mL of organic solvent (dichloromethane) was added to each sample tube and sealed with a cap to prevent the evaporation of DMA and organic solvent. The mixtures were vortexed (3 min) and sonicated for 30 min at a low temperature (0–4 °C) to release the entrapped amines, and then frozen for 10 min. Then, the samples were centrifugated for 3 min at 6000 rpm to achieve complete solid–liquid separation. Thus, in each tube, a precipitate was formed containing the insoluble substances in the organic solvent (excipients and API) and the supernatant, which was a clear solution of DMA and DEA.

**Figure 4:**
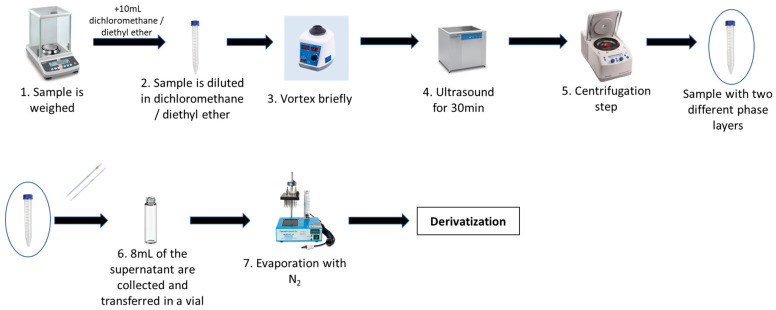
Sample preparation steps.

After collecting 8 mL of the supernatant, 200 µL H_2_O (prevents DMA evaporation) was added, and the samples were vortexed (1 min) and gently evaporated (with N_2_, at low temperature) to remove dichloromethane (up to 200 µL) (Figure 4).

Finally, in the subsequent derivatization stage, 150 μL of NBD-CL (concentration = 0.33 mg/mL), 50 μL of borate buffer solution (20 mM, pH = 11), and 200 μL of the sample (final concentrations: DMA = 3 ng/mL and DEA 30 ng/mL) were reacted. To remove sediment, the samples were filtered with a 0.45 μm PTFE filter prior to their analysis by HPLC. To select the filter with the best performance, a sample with final concentrations of 3 ng/mL DMA and 30 ng/mL DEA was filtered with four different types of filters (0.45 μm), and their performances were compared (Table S5).

To check the accuracy of the extraction process, five samples (0.12 ppm DMA and 1.2 ppm DEA) and two blanks (the first after the derivatization process and the second after the purification and derivatization process) were tested for each preparation (spike and un-spike). The recoveries of both DMA and DEA were calculated against the reference standard (mixed STD1) and are shown in Table 6.

#### 2.4.2. Solid-Phase Extraction (SPE)

Several problems had to be overcome during sample processing in order to achieve complete purification and quantitative recovery of DMA and DEA. Mainly, it was desired that the derivatization reaction with NBD-Cl would be carried out selectively only with DMA and DEA and not with the rest of the substrate (excipients, API, impurities). However, due to the complexity of the samples, such a process with the liquid extraction technique was laborious and would have had reduced efficiency. Alternatively, considering that both amines were small, volatile, polar, and water-soluble molecules (unlike the rest of the substrate), the problem was addressed by applying a C_18_ SPE technique. The novelty of the present SPE purification is in its execution in contrast to the established procedure, which can be described as follows: 600 μL of water was added to 25 mg of substrate, and the sample was alternately sonicated and vortexed for 15 min. The sample was then loaded onto an SPE Bond Elute-C18 cartridge (washed with 1 mL MeOH, conditioning with 2 mL H_2_O) where both amines, as small polar molecules, were co-eluted with water. After the eluate was collected, 200 μL of it was subjected to derivatization. To confirm complete elution of both substances, the cartridge was washed with another 400 µL of water in which no traces of amines were detected.

Sample purification was tested using two different SPE C18, 500 mg 6 ml cartridges (Bond Elute, by Agilent Technologies (Santa Clara, CA, USA) and Suplelclean^TM^ ENVITM by Supelco (St. Louis, MO, USA)). The Suplelclean^TM^ ENVITM cartridge was rejected because it gave interfering peaks at the same retention time as that of DMA. Quantification of the samples (Table 6) was performed based on a reference calibration curve where standard solutions were subjected to the same clean-up steps as those of the proposed SPE procedure (Table 3). Because the values of matrix effects (ΜΕ) were within acceptable limits (Uniphyllin^®^: %ME_DMA_ = 106.7, %ME_DEA_ = 85.0, Xylosan^®^: %ME_DMA_ = 97.9, %ME_DEA_ = 85.7), and to simplify the method, a calibration curve from spiked standard solutions on a substrate was not used.

For the recovery study, five samples for each preparation (spike and un-spike) and two blanks (one with diluent and one with diluent subjected to purification and derivatization) were utilized. Substrate spike samples were prepared by adding 200 μL STD1 mix to 25 mg formulation (0.5 ppm DMA and 5 ppm DEA).

**Figure 5:**
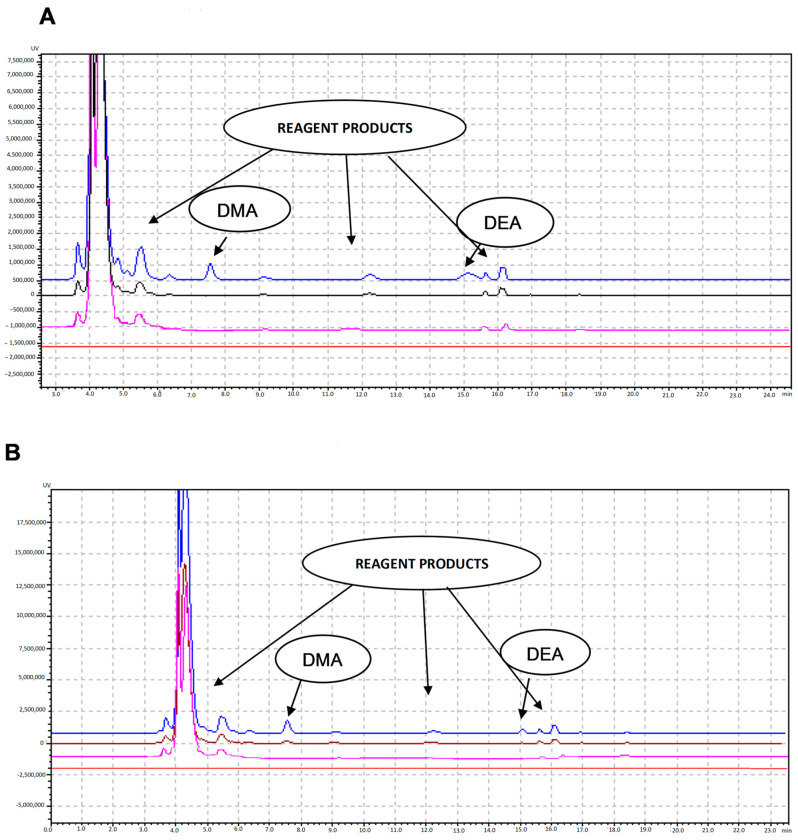
Chromatograms of (**A**) blank diluent (red), blank after SPE and derivatization (pink), unspike for Uniphyllin tablets (black) and spiked for Uniphyllin tablets (blue); (**B**) blank diluent (red), blank after SPE and derivatization (pink) unspike for Xylosan 2% (brown) and spiked for Xylosan 2% (blue).

According to the results, there was no DMA or DEA in Uniphyllin^®^ tablets, while in Xylosan^®^ 2%, only 0.019 µg/mL DMA was found (Figure 5A,B).

## 3. Materials and Methods

### 3.1. Chemicals and Solutions

All chemicals used were of analytical grade (Purity ≥ 99%). The reagents boric acid, NaOH in pellets, and dichloromethane (DCM) were obtained from Merck (Darmstadt, Germany), while H_3_PO_4_ (≥85%) was purchased from Honeywell Fluka (Durham, NC, USA) and formic acid from Scharlau (Aarau, Switzerland). Methanol was of HPLC-grade purity and was obtained from VWR Chemicals (Radnor, PA, USA). Water was ultrapure for HPLC (18.2 MΩ cm resistivity) and was produced using a purification system (B30, Adrona SIA, Riga, Latvia). The pharmaceutical formulations (Uniphyllin^®^ tablets and Xylozan^®^ solution 2%) were purchased from a local pharmacy.

The 4-chloro-7-nitrobenzofurazane derivatization reagent solution was prepared by dissolving 33.0 mg of NBD-Cl in 100 mL of MeOH. The solution was stored at 2–8 °C in dark vials. To prepare the borate buffer (20 mΜ, pH 11), 122 μg of boric acid was dissolved in 100 mL of water, and the pH of the solution was adjusted using a concentrated solution of NaOH (1.0 M). Phosphate buffer (20 mM, pH 2.8) was prepared by adding 2.759 g of NaH_2_PO_4_ to 1 lt of water. The pH of the phosphate buffer was adjusted using phosphate acid.

### 3.2. Standards Solutions

Stock solutions of DMA and DEA were prepared as follows: 10 mg of DMA and DEA were weighed and dissolved with MeOH in two separate 10 mL volumetric flasks (1 mg/mL). Then, 1 mL DEA and 0.1 mL DMA were quantitatively transferred into the same 10 mL volumetric flask and filled with methanol (0.1 mg/mL DEA and 0.01 mg/mL DMA). Next, 1 mL of the mixture was transferred to a 50 mL flask and filled to the mark with H_2_O. Finally, two series of six standard solutions were made to prepare two different calibration curves (Table 3). For the first, 200 μL of each standard solution, after undergoing derivatization, was analyzed by HPLC-FLD. For the reference calibration curve, 600 µL of each standard solution was subjected to the SPE procedure, and 200 µL of the eluate was analyzed after derivatization.

### 3.3. Instrumentation and Chromatographic Conditions

Chromatographic separations were performed using a Shimadzu HPLC system consisting of two LC-20AD pumps, a DGU 14A degasser, a SIL-10AD autosampler, and an RF20-A fluorescence detector (Shimadzu, Tokyo, Japan). NBD derivatives were detected at _λex/λem= 450/540 nm. The analytical column was a reversed-phase LC-C18 HS (250 × 4.6 mm), 5.0 μm, Supelco, thermostatically controlled with a CTO-10AS VP heated chamber (Shimadzu, Tokyo, Japan) at 40 °C.

The mobile phase contained two solvents (Eluent A: NaH_2_PO_4_, 20 mM, pH = 2.8 and Eluent B: MeOH) which were gradient-eluted (maintained up to 6 min in 50% eluent B, rose from 9–14 min to 80%, and returned in the next 4 min to 50% B), with a flow rate of 0.8 mL/min (Table S6). Data processing was carried out using the LC solution software version 1.25 SP4.

### 3.4. Derivatization Procedure

Briefly, 200 μL of each standard solution, 50 μL of borate buffer (20 mM), and 150 μL of NBD-CL (0.33 mg/mL) were quantitatively transferred to a vial and mixed by vortexing for 1 min. The sample was then placed in a heating block at 70 °C for 30 min and in the freezer for 1 min to stop the reaction. After the completion of the reaction, each sample was analyzed by HPLC-FLD.

### 3.5. Solid Phase Extraction

First, 600 µL of water was added to 25 mg of substrate (Uniphyllin^®^, Xylozan^®^), and the sample was alternately sonicated and vortexed for 15 min. Bond Elute (500 mg, 6 mL) was then loaded onto a SPE-C18 cartridge (washed with 1 mL MeOH, conditioned with 2 mL H_2_O). After collecting the eluate, 200 µL of it was subjected to derivatization.

## 4. Conclusions

The present study proposes a simple and cost-effective analytical approach with HPLC and FLD detectors for the quantitative determination of dimethylamine (DMA) and diethylamine (DEA) in pharmaceutical formulations. Both substances were derivatized using NBD-Cl reagent and borate buffer at pH = 11. For the analysis of the two complexes, NBD-DMA and NBD-DEA, the optimal chromatographic conditions were determined, and the proposed method was validated according to ICH regulations. Purification of the samples (Uniphyllin^®^ tablets and Xylozan^®^ 2% solution) was achieved using a one-step SPE technique, which was efficient, since their recoveries were in the range of 81.7–88.8% for DMA (0.5 ppm) and 81.1–95.8% for DEA (5 ppm).

## Figures and Tables

**Table 1:** Crossed D-Optimal Experimental Design (CDO) and parameter limits used.

Study Type	Combined			Runs	18	
Design Type	D-optimal			Blocks	No Blocks	
Design Model	Quadratic × Quadratic					
Mixture	Components	A	Vof NBD-Cl (μL)	Mixture	50	150
		B	V of water/DMA-DEA (μL)	Mixture	150	250
		A + B =				400
	Process Factors	C	Volume of borate buffer (μL)	Numeric	50	200
		D	Time (min)	Numeric	10.0	80.0

**Table 2:** Statistical analysis (ANOVA) of CDO at 95% confidence level for the following responses: (**a**) AREA of DMA and (**b**) AREA of DEA.

**Source**	**Sum of Squares**	**df**	**Mean Square**	**F-Value**	***p*-Value**
Model	1.21	4	3.044	51.8	<0.0001
^(1)^Linear Mixture	3.005	1	3.005	51.13	<0.0001
AD	5.05	1	5.05	85.93	<0.0001
BC	9.512	1	9.512	16.19	0.0014
BD	1.339	1	1.339	22.79	0.0004
Residual	7.64	13	5.887		
Cor Total	1.294	17			
	Standard Deviation	7.666	R^2^	0.941	
	Mean	4.304	Adjusted R^2^	0.9228	
Fit Statistics	C.V.%	17.81	Predicted R^2^	0.8743	
			Adequate Precision	24.0773	
(a)					
**Source**	**Sum of Squares**	**df**	**Mean Square**	**F-Value**	***p*-Value**
Model	8.86	6	1.477	65.78	<0.0001
^(1)^Linear Mixture	6.339	1	6.339	282.41	<0.0001
AC	9.905	1	9.905	44.13	<0.0001
AD	3.564	1	3.564	15.88	0.0021
BC	1.1	1	1.1	4.9	0.0489
ACD	1.845	1	1.845	8.22	0.0153
AD^2^	8.338	1	8.338	37.15	<0.0001
Residual	2.469	11	2.245		
Cor Total	9.107	17			
	Standard Deviation	47,377.83	R^2^	0.9729	
	Mean	3.116	Adjusted R^2^	0.9581	
Fit Statistics	C.V.%	15.2	Predicted R^2^	0.9181	
			Adequate Precision	25.4447	
(b)					

**Table 3:** The concentrations and parameters of the standards associated with the calibration curves.

**Agents**	**Concentration Range** **(ng/mL)**	**Calibration Curve**	**Correlation Coefficient**	**Limit of Detection (ng/mL)**	**Limit of Quantitation** **(ng/mL)**
DMA	0.5–10	y = 49,530x + 12,828	0.998	0.1	0.4
DEA	5–100	y = 3213.2x − 5063	0.999	0.9	3
**Agents**	**Concentration Range** **(ng/mL)**	**Reference Calibration Curve (After SPE)**	**Correlation Coefficient**	**Limit of Detection (ng/mL)**	**Limit of Quantitation** **(ng/mL)**
DMA	1.7–20	y = 28,322x + 41,357	0.997	0.3	1.3
DEA	16.7–200	y = 3162.8x – 18,137	0.998	3.0	10.0

**Table 4:** The % recovery values of DMA and DEA.

Concentration DMA (ng/mL)	Found Concentration DMA (ng/mL)	Recovery (%)	Concentration DΕA (ng/mL)	Found Concentration DΕA (ng/mL)	Recovery (%)
0.5	0.51	102	5	4.91	98.2
2.5	2.54	101.6	25	25.4	101.6
4.38	4.385	99.8	43.75	43.70	99.9
6	5.94	99	60	60.6	101
10	10.01	100.1	100	99.8	99.8

**Table 5:** Intra- and inter-day precision.

Agents	Repeatability		Intermediate Precision				
	Concentration (ng/mL)	%RSD	Concentration (ng/mL)	%RSD			
				1st Day	2nd Day	3rd Day	Total
DΜA	0.5 (n = 5)	2.7	0.5 (n = 5)	2.7	1.1	2.8	1.7
	5 (n = 3)	0.2	5 (n = 3)	0.2	0.1	0.6	1.9
	10 (n = 3)	0.3	10 (n = 3)	0.3	0.8	0.4	0.5
DΕA	5 (n = 5)	2.4	5 (n = 5)	2.4	0.3	2.3	2.5
	50 (n = 3)	2.2	50 (n = 3)	2.2	0.6	0.5	2.9
	100 (n = 3)	0.3	100 (n = 3)	0.3	1. 9	0.4	1.9

**Table 6:** Sample recoveries of 5 repetitions using LE and SPE procedures.

	**Liquid Extraction**			
	% Recovery DMA	%RSD	% Recovery DEA	%RSD
Ref. Standard	77.1	2.0	68.9	2.0
Uniphyllin	107.8	4.8	65.7	9.1
Xylosan	65.3	3.0	95.6	3.3
	**Solid Phase Extraction**			
Standard	83.31	0.57	95.8	0.7
Uniphyllin	88.83	2.98	81.1	4.9
Xylosan	81.58	0.42	82.1	0.9

## Data Availability

Data are contained within the article and Supplementary Materials.

## Supplementary Materials

The following supporting information can be downloaded at: https://www.mdpi.com/article/10.3390/molecules29235535/s1, Table S1. Results derived from toxicity tests of DMA, DEA; Table S2. Effect of derivatization temperature on signal intensity; Figure S1. Chromatogram of the diluted mixed DEA and DME standard with addition of borate buffer with pH = 9 (blue), pH = 10 (black), and pH = 11 (pink); Figure S2. Chromatogram of the diluted mixed DEA and DME standard with addition of borate buffer with C = 5 mM (blue), C = 10 mM (black), and C = 20 mM (pink); Figure S3. (A) Plot of residuals vs. predicted values for area of DEA and DMA, (B) actual vs predicted values; Table S3. Final equations for two responses (area of DMA and DEA) in terms of real component and actual factors; Figure S4. Derivatization steps; Figure S5. Chromatogram of the diluted DEA and DMA standard mix with Eluent A formic acid (black) and phosphoric acid (pink) buffer solution (pH = 2.8, 20 mM). Composition of mobile phase: Eluent A/Eluent B = 50/50%; Figure S6. Fluorescence emission (left) and excitation (right) scan; Table S4. Changes in parameters and robustness investigation; Table S5. % filter recoveries for DMA and DEA; Figure S7. Investigation of liquid extraction; Table S6. Gradient elution program of the mobile phase.
