# Supplementary material for: A Pre-Column Derivatization Method for the HPLC-FLD Determination of Dimethyl and Diethyl Amine in Pharmaceuticals
Source: Molecules. 2024 Nov 23;29(23):5535. doi: 10.3390/molecules29235535 (PMC11643484; doi:10.3390/molecules29235535)
Supplement: Supplementary file 1 [file molecules-29-05535-s001.zip › molecules-3316177-supplementary.pdf]

# Supplementary Materials

# A pre-column derivatization method for the HPLC-FLD determination of dimethyl and diethyl amine in pharmaceuticals

**Georgios Kamaris [1], Maria Tsami [1], Georgiana-Roxana M. Lotca[1], Sofia Almpani[1] and Catherine K. Markopoulou [1,*]**

[1] Laboratory of Pharmaceutical Analysis, Department of Pharmacy, Aristotle University of Thessaloniki,54124 Thessaloniki, Greece; kamarisg@pharm.auth.gr ; tsamimaria@gmail.com; grlotca@gmail.com ; salmpan@pharm.auth.gr; amarkopo@pharm.auth.gr

* Correspondence: amarkopo@pharm.auth.gr ; Tel.: +30 2310 997665

**Table S1.** Results derived from toxicity tests of DMA, DEA.

| ID | Value DMA | Value DEA |
| --- | --- | --- |
| Ames_test | Non-mutagen | mutagen |
| Carcino_Mouse | positive | positive |
| Carcino_Rat | positive | positive |
| hERG_inhibition | medium_risk | medium_risk |
| *TA100_10RLI | negative | negative |
| *TA100_NA | negative | negative |
| *TA1535_10RLI | negative | negative |
| *TA1535_NA | negative | positive |

* strains of Salmonella typhimurium and E.coli

**Table S2.** Effect of derivatization temperature on signal intensity

| Temperature (° C) | Area DMA | Area DEA |
| --- | --- | --- |
| 25 | 158841 | 41570 |
| 40 | 344564 | 154622 |
| 70 | 518209 | 321944 |
| 80 | 500237 | 342641 |
| 100 | - | - |

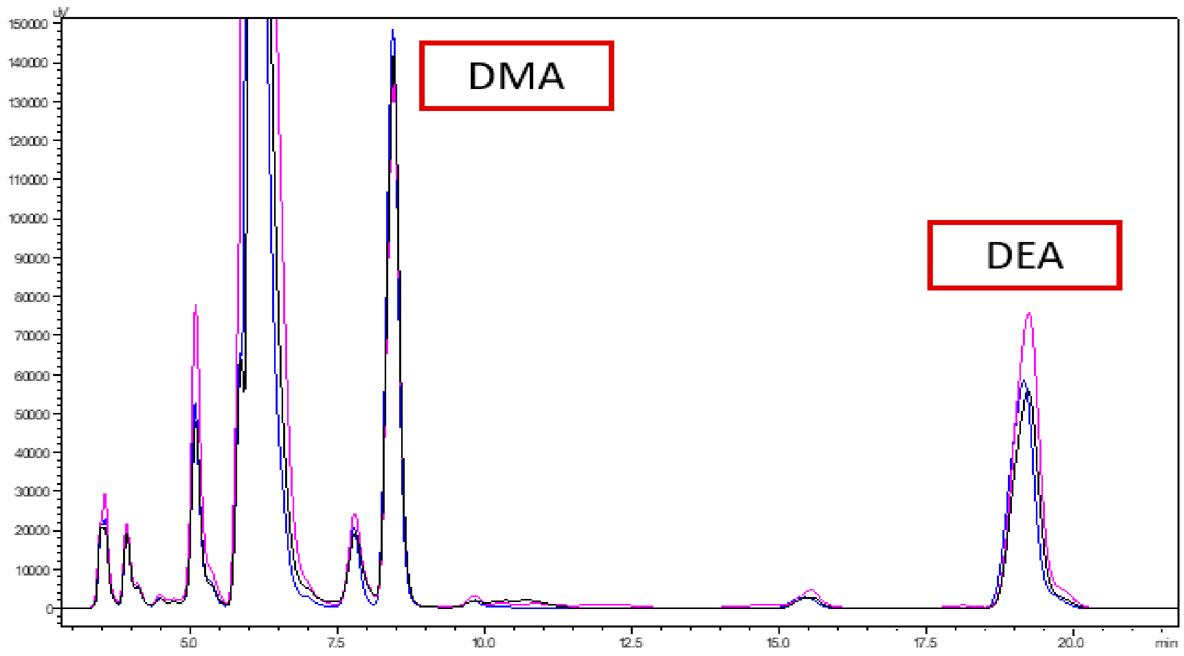


**Figure S1.** Chromatogram of the diluted mix DEA and DME standard with addition of borate buffer with pH=9 (blue), pH=10 (black) and pH=11 (pink).

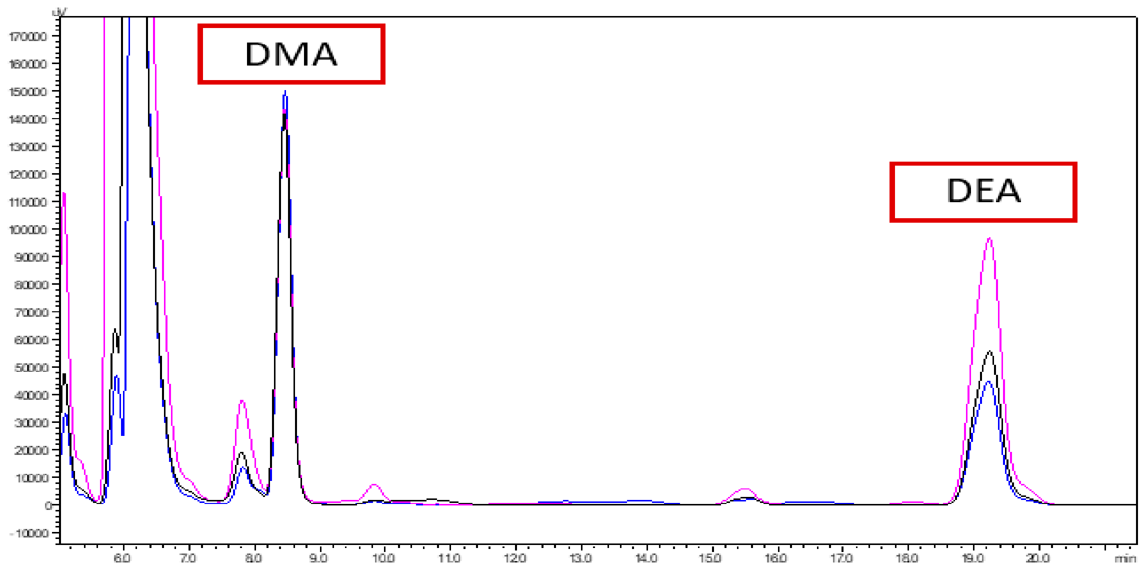


**Figure S2**. Chromatogram of the diluted mix DEA and DME standard with addition of borate buffer with C=5mM (blue), C=10mM (black) and C=20mM (pink).

## Response 1.Normalized Area DMA

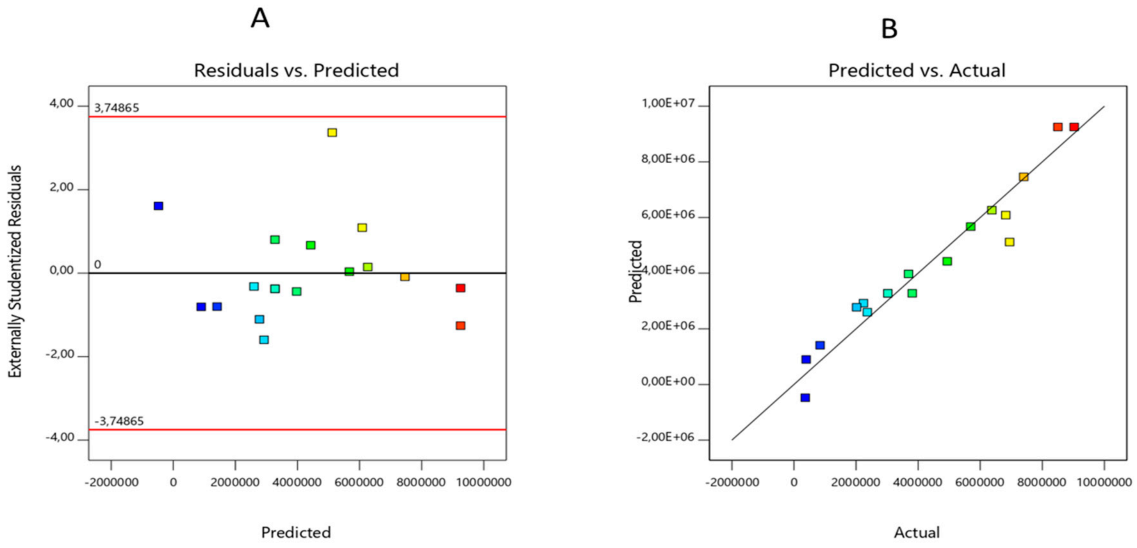


## Response 2.Normalized Area DEA

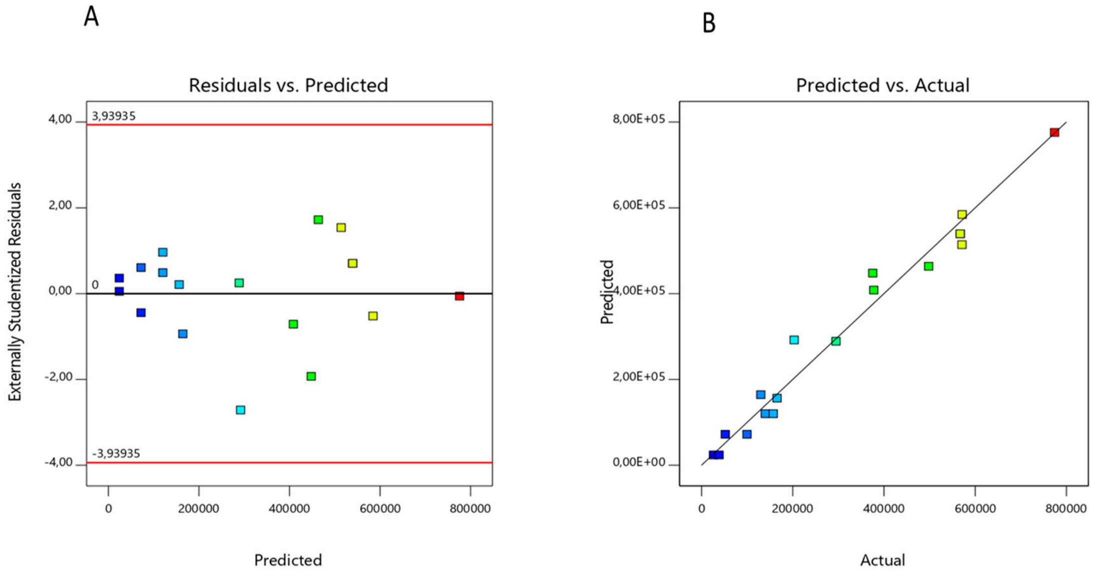


**Figure S3** : (A) Plot of residuals vs predicted values for Area of DEA and DMA, (B) Actual vs predicted values

**Table S3.** Final equations for two Responses (Area of DMA and DEA)in terms of real component and actual factors

| Normalized Area DEA |
|---|
| +5.83355E+05 V (NBD) |
| +1.43201E+ 05 V ($H_2O$) |
| -1865.44154 V (NBD) * V buffer |
| +74755.45712 V (NBD) * time T |
| - 782.00842 V ($H_2O$) * V buffer |
| -14951.09142 V ($H_2O$) * time T |
| -106.31840 V (NBD) * V buffer * time T |
| +21.26368 V ($H_2O$) * V buffer * time T |
| -588.69014 V (NBD) * time $T^2$ |
| +117.73803 V ($H_2O$) * time $T^2$ |

| Normalized Area DMA |
|---|
| +1.19005E+07 V (NBD) |
| +831062E+06 V ($H_2O$) |
| +41259.31204 V (NBD) * V buffer |
| -140589E+ 05 V (NBD) * V buffer |
| -41259.31204 V ($H_2O$) * V buffer |
| -30142.69391 V ($H_2O$) * time T |

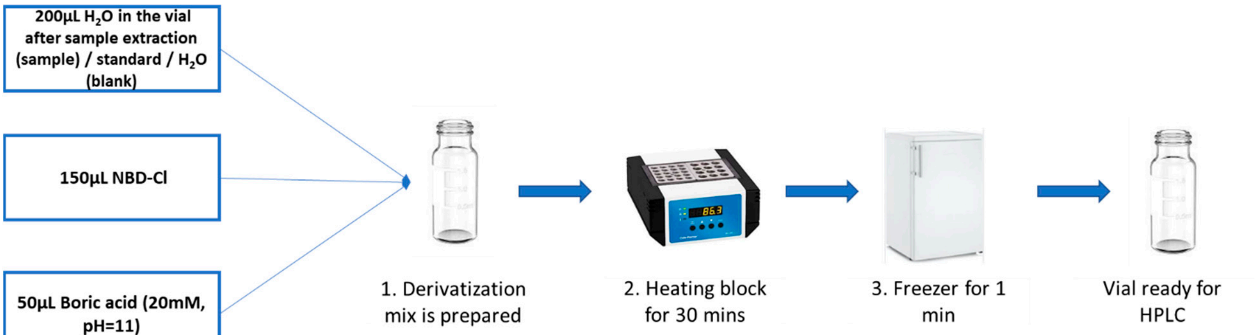


**Figure S4**. Derivatization steps

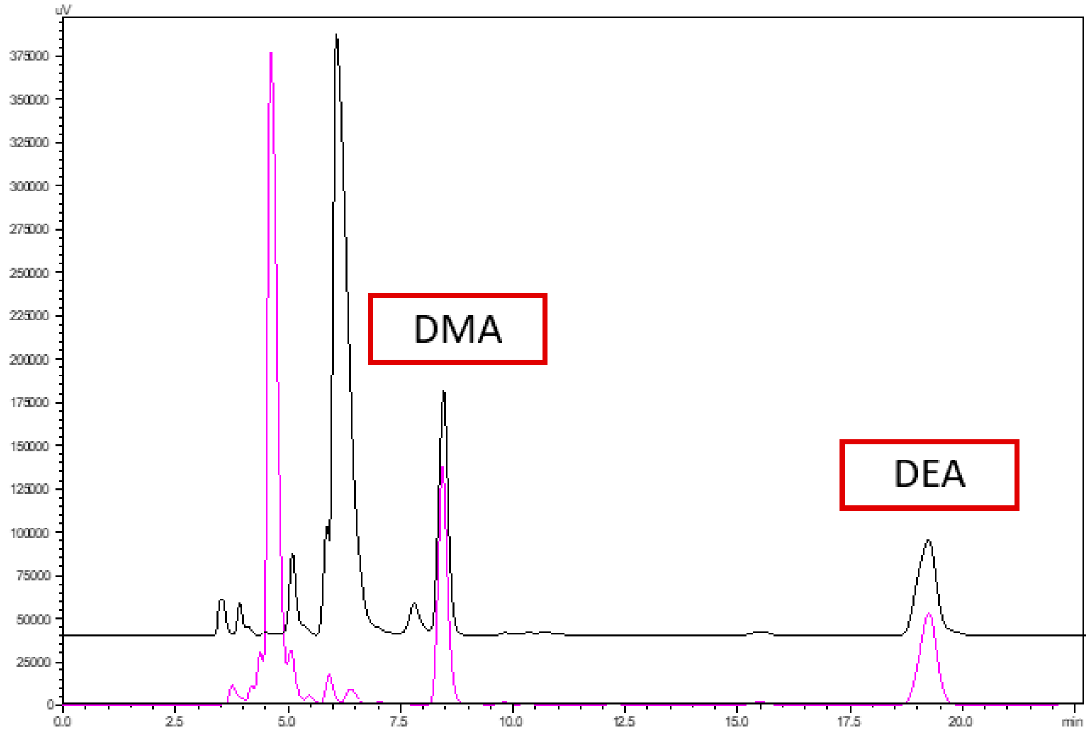


**Figure S5.** Chromatogram of the diluted DEA and DMA standard mix with Eluent A formic acid (black) and phosphoric acid (pink) buffer solution (pH=2.8, 20mM). Composition of mobile phase: Eluent A / Eluent B = 50 / 50 %.

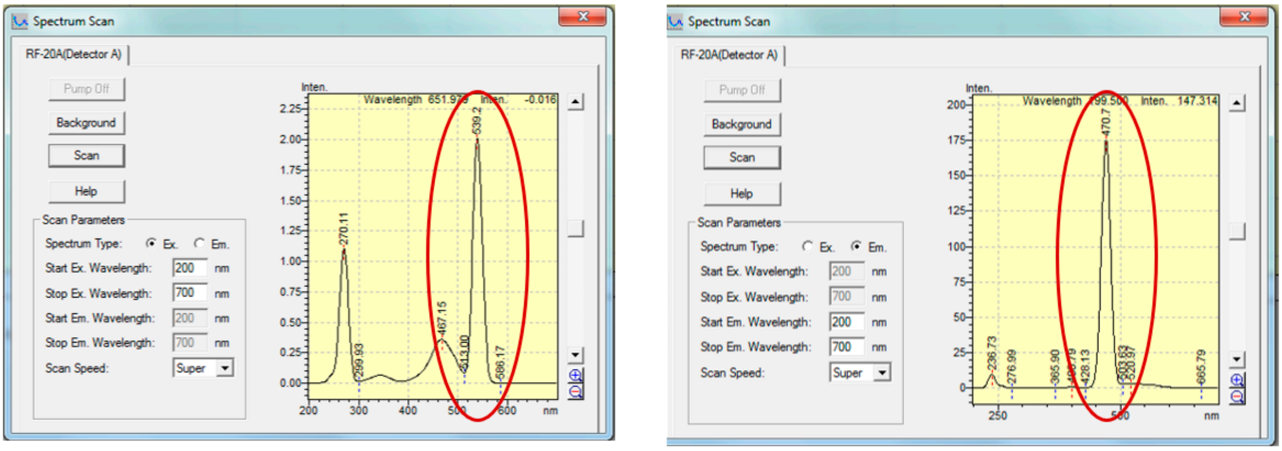


**Figure S6.** Fluorescence emission (left) and excitation (right) scan.

**Table S4.** Changes of parameters and robustness investigation.

| Parameters | %RSD of Peak Area for DMA | %RSD of Peak Area for DEA | %RSD of tailing factor for DMA | %RSD of tailing factor for DEA | % RSD Resolution Factor with the nearest peak for DMA | % RSD Resolution Factor with the nearest peak for DEA |
|---|---|---|---|---|---|---|
| Mobile phase A:B (50:50,49:51,51:49) | 0.7% | 2.2% | 0.6% | 1.8% | 1.9% | 0.5% |
| Flow Rate mL/min (0.8, 0.82, 0.78) | 1.7% | 1.7% | 0.5% | 0.4% | 0.7% | 1.8% |
| Column T (°C) (40,39,41) | 1.9% | 2.0% | 0.1% | 0.7% | 2.0% | 1.7% |
| $\lambda_{ex/em}$ (450/540, 451/541, 449/539) | 1.0% | 1.,0% | 0.0% | 0.4% | 0.7% | 0.1% |

**Table S5**. % filter recoveries for DMA and DEA

| **Filter** | **DMA** | | **DEA** | |
|---|---|---|---|---|
| | **Area** | **% Recovery** | **Area** | **% Recovery** |
| CA | 472088 | 91.1 | 263994 | 82 |
| PTFE | 489729 | 94.5 | 292331 | 90.8 |
| PVD | 486598 | 93.9 | 256267 | 79.6 |
| Nylon | 495926 | 95.7 | 209585 | 65.1 |

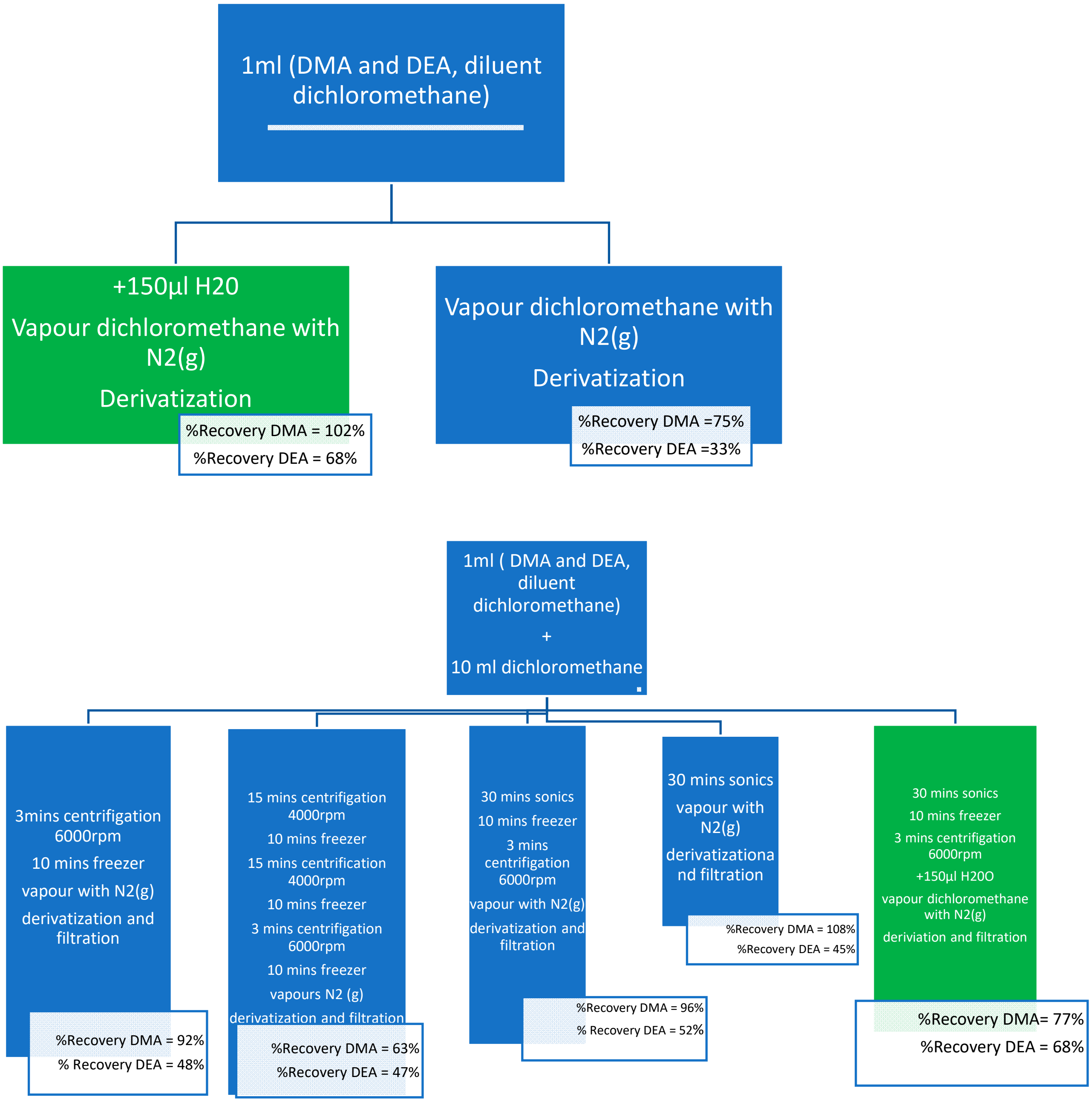


**Figure S7.** Investigation of Liquid Extraction

P.S. At procedures with addition of H2O before vaporization with N2(g). the derivatization happens with the non-vapored H20 (150µL) and 150 µL NBD-Cl and 50µL borate buffer. At the any other circumstance. the derivatization with 150µL H20. 150 µL NBD-Cl and 50 µL borate buffer.

**Table S6**. Gradient elution program of the mobile phase

P.S. Pump A: Phosphoric Acid Buffer (20 mM, pH= 2.8) and Pump B: Methanol

| Time | Concentration of B |
| --- | --- |
| 6.00 | 50 |
| 9.00 | 80 |
| 14.00 | 80 |
| 18.00 | 50 |
| 27.00 | 50 |
| 27.00 | Controller Stop |
